# A multicenter randomized phase III trial of weekly cisplatin chemoradiotherapy versus radiotherapy in elderly patients with head and neck squamous cell carcinoma unfit for high-dose cisplatin: JCOG2405 (ELDER-WEEKLY)

**DOI:** 10.1093/jjco/hyag097

**Published:** 2026-06-24

**Authors:** Kota Kawabata, Koichi Yasuda, Satoaki Nakamura, Yoshitaka Honma, Ryusuke Suzuki, Yuri Ueda, Satoshi Saito, Kazuto Matsuura, Nobuhiro Hanai, Naoki Nakamura, Junki Mizusawa, Keita Sasaki, Haruhiko Fukuda, Takashi Mizowaki, Naomi Kiyota, the Head and Neck Cancer Study Group and the Radiation Therapy Study Group of the Japan Clinical Oncology Group

**Affiliations:** Japan Clinical Oncology Group Data Center/Operations Office, National Cancer Center Hospital, Tokyo, 5-1-1 Tsukiji, Chuo-ku, Tokyo 104-0045, Japan; Department of Radiation Oncology, Hokkaido University Hospital, North-15 West-7, Sapporo, Hokkaido 060-8638, Japan; Division of Radiation Oncology, Kansai Medical University, 2-5-1 Shinmachi, Hirakata, Osaka 573-1010, Japan; Department of Head and Neck, Esophageal Medical Oncology, National Cancer Center Hospital, 5-1-1 Tsukiji, Chuo-ku, Tokyo 104-0045, Japan; Department of Medical Physics, Hokkaido University Hospital, North-15 West-7, Sapporo, Hokkaido 060-8638, Japan; Department of Otorhinolaryngology, Head and Neck Surgery, Tokyo Medical University, 6-7-1 Nishishinjuku, Shinjuku-ku, Tokyo 160-0023, Japan; Department of Radiation Oncology, National Cancer Center Hospital East, 6-5-1 Kashiwanoha, Kashiwa, Chiba 277-8577, Japan; Department of Head and Neck Surgery, National Cancer Center Hospital East, 6-5-1 Kashiwanoha, Kashiwa, Chiba 277-8577, Japan; Department of Head and Neck Surgery, Aichi Cancer Center Hospital, 1-1 Kanokoden, Chikusa-ku, Nagoya, Aichi 464-8681, Japan; Department of Radiation Oncology, St. Marianna University School of Medicine, 2-16-1 Sugao, Miyamae-ku, Kawasaki, Kanagawa 216-8511, Japan; Japan Clinical Oncology Group Data Center/Operations Office, National Cancer Center Hospital, Tokyo, 5-1-1 Tsukiji, Chuo-ku, Tokyo 104-0045, Japan; Japan Clinical Oncology Group Data Center/Operations Office, National Cancer Center Hospital, Tokyo, 5-1-1 Tsukiji, Chuo-ku, Tokyo 104-0045, Japan; Japan Clinical Oncology Group Data Center/Operations Office, National Cancer Center Hospital, Tokyo, 5-1-1 Tsukiji, Chuo-ku, Tokyo 104-0045, Japan; Department of Radiation Oncology and Image-Applied Therapy, Graduate School of Medicine, Kyoto University, Yoshida Konoe-cho, Sakyo-ku, Kyoto 606-8501, Japan; Department of Medical Oncology and Hematology, Cancer Center, Kobe University Hospital, 7-5-2 Kusunoki-cho, Chuo-ku, Kobe, Hyogo 650-0017, Japan

**Keywords:** head and neck cancer, weekly cisplatin chemoradiotherapy, radiotherapy, elderly patients, unfit for cisplatin

## Abstract

Chemoradiotherapy (CRT) with high-dose cisplatin (100 mg/m^2^) is the standard treatment for locally advanced squamous cell carcinoma of the head and neck (SCCHN). However, this regimen is often intolerable in elderly patients because of its toxicity, and the optimal treatment for those unfit for high-dose cisplatin remains unclear. CRT with weekly cisplatin (40 mg/m^2^) has been developed as a more tolerable alternative and has demonstrated comparable efficacy with reduced toxicity in several trials involving nonelderly patients. We therefore designed a multicenter, randomized phase III trial (JCOG2405) to confirm the superiority of CRT with weekly cisplatin over radiotherapy alone in patients aged ≥70 years with locally advanced SCCHN who are unfit for high-dose cisplatin. The primary endpoint is overall survival. A total of 250 patients from 59 institutions will be enrolled over 5 years. The trial has been registered in the Japan Registry of Clinical Trials (study number: jRCTs031250688).

## Teaser

This multicenter, randomized phase III trial aims to confirm the superiority of weekly cisplatin-based chemoradiotherapy over radiotherapy alone in elderly patients with head and neck cancer unfit for high-dose cisplatin.

## Introduction

Head and neck cancer is relatively rare but has been steadily increasing in Japan [1]. Squamous cell carcinoma is the most common histological type and accounts for ~90% of head and neck cancers. With the rapid aging of the Japanese population, the incidence among elderly patients has increased markedly over the past two decades. According to the 2021 National Cancer Registry, more than half of newly diagnosed patients with head and neck cancer were aged ≥70 years [2]. A similar trend has been observed worldwide, with the global population of older adults expected to more than double over the next 30 years compared with that in 2019 [3]. The growing number of elderly patients with head and neck cancer has made the optimization of treatment strategies an important global issue [4].

For patients with locally advanced squamous cell carcinoma of the head and neck (SCCHN) who are not amenable to surgery—either because of unresectable disease or a preference for organ preservation despite resectability—the standard treatment is chemoradiotherapy (CRT) with concurrent 3 weekly high-dose cisplatin (CDDP) at 100 mg/m^2^ [5]. This regimen has long been recognized as the global standard based on several phase III randomized controlled trials and meta-analyses [6–10]. However, compared with younger patients, elderly patients generally have poorer overall condition and a higher burden of comorbidities [11–16], and CRT with high-dose CDDP is often difficult to tolerate because of severe adverse events (AEs), including nephrotoxicity, ototoxicity, and hematologic toxicity [17, 18].

In terms of efficacy, a meta-analysis integrating 107 randomized controlled trials (MACH-NC) demonstrated that, in the overall population with locally advanced SCCHN, CRT significantly improved overall survival (OS) compared with radiotherapy (RT) alone (hazard ratio [HR] = 0.83; 95% confidence interval [CI], 0.79–0.86). However, no clear survival benefit was observed in patients aged ≥70 years (HR = 0.97; 95% CI 0.91–1.16) [10]. Similar findings have been reported in several other analyses [8, 9] and the United Kingdom National Multidisciplinary Guidelines recommend RT alone as the standard treatment for this age group [19]. In contrast, the NCCN Guidelines and the International Society of Geriatric Oncology Guidelines recommend that treatment decisions should not be based solely on chronological age; rather, elderly patients with good overall condition and preserved organ function should be considered for the same treatment options as younger patients [12, 20]. In clinical practice, treatment strategies vary considerably across regions and institutions worldwide, and a unified consensus has not yet been established [21]. In Japan, CRT with high-dose CDDP is administered to elderly patients with SCCHN when feasible; for patients deemed unable to tolerate this regimen, alternative treatment strategies are employed, such as RT alone or CRT with less toxic chemotherapy regimens. In a nationwide survey conducted by the Head and Neck Cancer Study Group (HNCSG) of the Japan Clinical Oncology Group (JCOG), approximately half of the patients unfit for CRT with high-dose CDDP received RT alone, whereas the remaining half were treated with less toxic CRT regimens, most commonly those incorporating weekly CDDP [22].

CRT with weekly CDDP (40 mg/m^2^) was developed as a more tolerable alternative to 3 weekly high-dose CDDP, and dose fractionation has been reported to reduce toxicity [23, 24]. Weekly CDDP-based CRT is widely used as standard treatment for cervical cancer [25], and its utility has also been reported in several studies in head and neck cancer. In JCOG1008, a randomized controlled trial comparing CRT with weekly CDDP versus 3 weekly high-dose CDDP in postoperative patients with SCCHN demonstrated noninferiority in OS and a reduction in AEs [26]. In the ConCERT trial, which enrolled patients eligible for definitive CRT, weekly CDDP demonstrated non-inferiority in locoregional control and fewer AEs compared with high-dose CDDP [27]. Furthermore, Ghosh-Laskar *et al.* demonstrated the superiority of CRT with weekly CDDP over RT alone in terms of locoregional control in a randomized controlled trial [28]. However, these trials predominantly enrolled non-elderly patients, and evidence in elderly populations remains limited. Although several retrospective studies have reported favorable clinical outcomes of CRT with weekly CDDP in elderly patients [29–31], no prospective study has confirmed its efficacy, and the optimal treatment strategy for elderly patients unfit for high-dose CDDP remains unclear.

Based on this background, we designed a multicenter, randomized phase III trial (JCOG2405: ELDER-WEEKLY) to evaluate whether CRT with weekly CDDP improves OS compared with RT alone in patients aged ≥70 years with locally advanced SCCHN who are not amenable to surgery and are unfit for CRT with high-dose CDDP (Fig. 1). Before initiating this trial, we conducted a questionnaire survey among institutions belonging to the JCOG-HNCSG regarding indications for CRT in elderly patients [22]. The survey confirmed that age, performance status (PS), renal function, and comorbidities are key factors in determining eligibility. Based on these findings, we defined the eligibility criteria for patients considered unfit for CRT with high-dose CDDP in this trial (Fig. 2). Furthermore, although human papillomavirus (HPV)-related oropharyngeal cancer generally has a more favorable prognosis than HPV-unrelated cancer, there is currently no established evidence supporting modification of standard treatment solely based on HPV positivity. Therefore, this trial includes patients with p16-positive oropharyngeal cancer at stage II who belong to the high-risk group (T1–3N2M0), as well as those with stage III disease, based on both prognosis and the current landscape of treatment development. For stage II disease, N2 status, defined as bilateral cervical lymph node metastases, is considered a clinically important high-risk factor. In addition, the clinical outcomes of RT alone in this subgroup are not necessarily favorable, supporting the need to evaluate treatment intensification. Accordingly, only patients with T1–3N2M0 were included. Patients with stage III disease are generally considered to require intensive multimodality treatment and are clinically comparable to other locally advanced head and neck cancer populations included in this trial.

**Figure 1:**
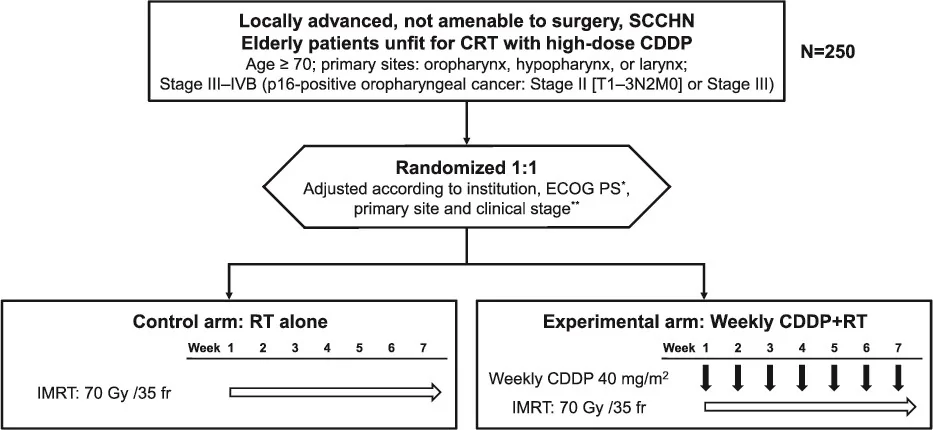
Study design. ^*^ECOG PS: 0 vs. 1–2; ^**^Primary site and clinical stage: p16-positive oropharyngeal cancer stage II or III vs. p16-negative oropharyngeal, hypopharyngeal, or laryngeal cancer stage III vs. p16-negative oropharyngeal, hypopharyngeal, or laryngeal cancer stage IVA–IVB. SCCHN, squamous cell carcinoma of the head and neck; CRT, chemoradiotherapy; CDDP, cisplatin; ECOG PS, Eastern Cooperative Oncology Group performance status; IMRT, intensity-modulated radiation therapy.

**Figure 2:**
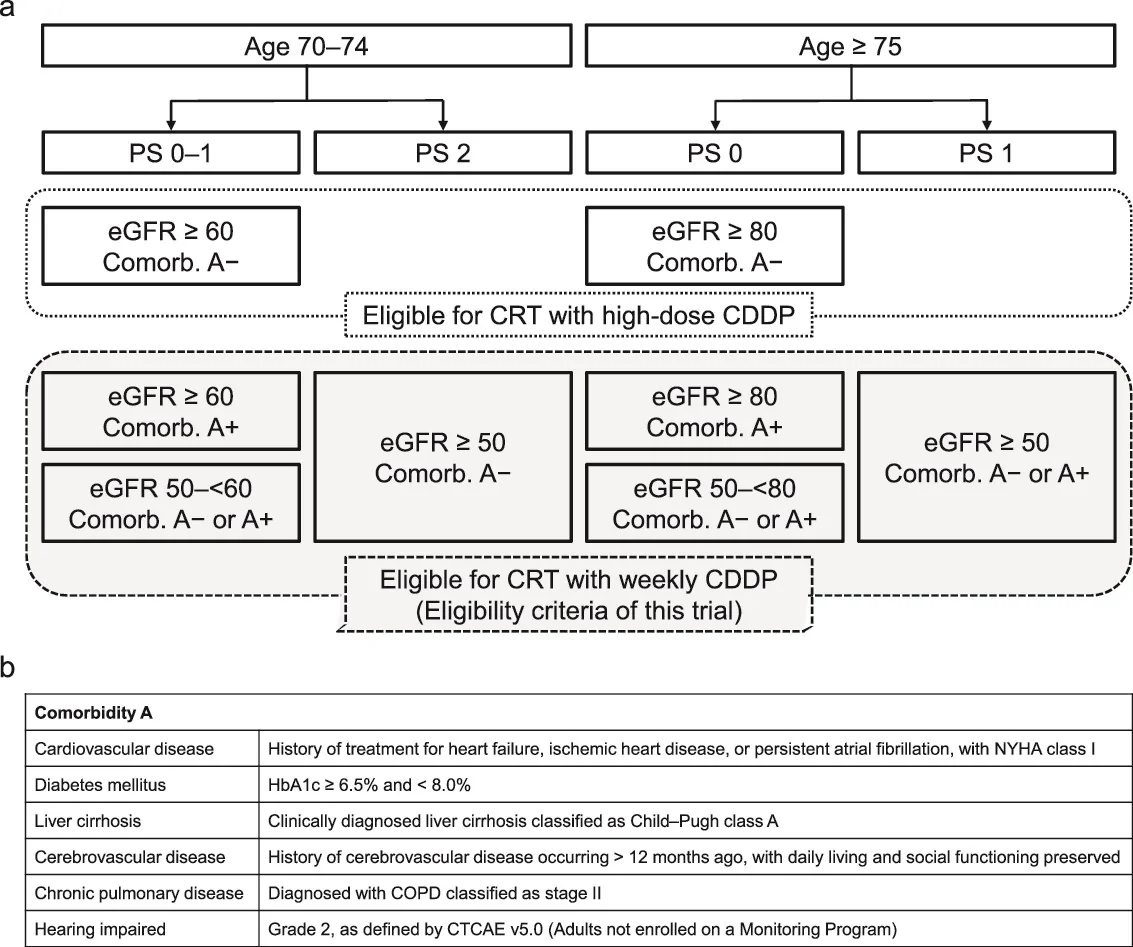
Target population. (a) Schema illustrating the elderly population eligible for this trial who are unfit for CRT with high-dose CDDP but may be eligible for CRT with weekly CDDP, based on age, PS, renal function, and comorbidities. Comorb. A+, presence of comorbidity A; Comorb. A−, absence of comorbidity A. (b) Details of comorbidity A. PS, performance status; CRT, chemoradiotherapy; CDDP, cisplatin; NYHA, New York Heart Association; COPD, chronic obstructive pulmonary disease.

This study protocol was approved by the Certified Review Board of the National Cancer Center Hospital in December 2025, and the trial was activated in January 2026. The trial was registered in the Japan Registry of Clinical Trials (jRCTs031250688; https://jrct.niph.go.jp/latest-detail/jRCTs031250688).

## Protocol digest of JCOG2405

### Purpose

We are conducting a randomized phase III trial to confirm the superiority of CRT with weekly CDDP compared with standard RT alone in elderly patients with locally advanced SCCHN who are not amenable to surgery and are unfit for CRT with high-dose CDDP.

### Study setting

This is a multicenter (59 specialized centers), open-label, randomized controlled phase III trial.

### Endpoints

The primary endpoint is OS in all randomized patients analyzed on an intent-to-treat basis. OS is defined as the time from randomization to death from any cause and is censored on the date of last contact for surviving patients. Secondary endpoints include progression-free survival (PFS), time to treatment failure (TTF) 1, TTF2, locoregional PFS, complete response proportion, cumulative incidence of locoregional progression, cumulative incidence of distant metastasis, cumulative incidence of disease-specific death, cumulative incidence of death from other causes, frequency of AEs, frequency of serious AEs, dose intensity of CDDP, proportion of protocol treatment completion, quality of life (QOL) scores, and the proportion of patients without deterioration in instrumental activities of daily living (IADL).

PFS is defined as the time from randomization to the earliest date of disease progression or death from any cause and is censored on the last contact date for patients alive without evidence of disease progression. TTF1 is defined as the time from randomization to disease progression, determination of residual disease after completion of protocol treatment, or death from any cause, whichever occurs first, and is censored on the last date the patient is alive without evidence of disease progression. In the definition of TTF2, clinical residual disease classified as a TTF1 event is not considered an event or censor if complete resection is conducted by salvage surgery and subsequent histopathological examination demonstrates a pathological complete response. TTF1 and TTF2 were used as endpoints in JCOG1912 [32], and the same definitions were adopted in this trial. AEs are evaluated according to the Common Terminology Criteria for Adverse Events version 5.0 (CTCAE v5.0). QOL is assessed using the EORTC QLQ-C30 and EORTC QLQ-HN43.

### Inclusion criteria

(1) Primary lesion located in the oropharynx, hypopharynx, or larynx.
(2) Histologically confirmed squamous cell carcinoma based on biopsy of the primary lesion. For oropharyngeal cancer, p16 status is confirmed by immunohistochemical staining.
(3) Clinical stage III–IVB for p16-negative oropharyngeal, hypopharyngeal, or laryngeal cancer, or stage II (T1–3N2M0) or stage III for p16-positive oropharyngeal cancer (UICC-TNM, 8th edition), based on the following evaluations:
  (i) Contrast-enhanced computed tomography (CT) or magnetic resonance imaging (MRI) of the head and neck
  (ii) Thoracoabdominal CT
(4) Aged ≥70 years.
(5) Eastern Cooperative Oncology Group (ECOG) PS 0–2.
(6) Patients meet any of the following criteria (i)–(vi) based on age, ECOG PS, renal function, and comorbidities (Fig. 2a).
^*^ Comorbidity A: cardiovascular disease, diabetes mellitus, liver cirrhosis, cerebrovascular disease, chronic pulmonary disease, or hearing impairment (Fig. 2b).
  (i) All of the following: (a) age 70–74 years, (b) PS 0–1, (c) eGFR ≥60 ml/min/1.73 m^2^, and (d) ≥1 comorbidity A^^*^^ present
  (ii) All of the following: (a) age 70–74 years, (b) PS 0–1, and (c) eGFR 50–<60 ml/min/1.73 m^2^
  (iii) All of the following: (a) age 70–74 years, (b) PS 2, (c) eGFR ≥50 ml/min/1.73 m^2^, and (d) no comorbidity A^^*^^
  (iv) All of the following: (a) age ≥ 75 years, (b) PS 0, (c) eGFR ≥80 ml/min/1.73 m^2^, and (d) ≥1 comorbidity A^^*^^ present
  (v) All of the following: (a) age ≥ 75 years, (b) PS 0, and (c) eGFR 50–<80 ml/min/1.73 m^2^
  (vi) All of the following: (a) age ≥ 75 years, (b) PS 1, and (c) eGFR ≥50 ml/min/1.73 m^2^
(7) Measurable lesions are not mandatory.
(8) All of the following:
  (i) No prior treatment with CDDP for any cancer.
  (ii) No prior RT to the brain or head and neck region.
  (iii) No prior surgery for cancers originating in the oropharynx, hypopharynx, or larynx.
  (iv) No prior neck lymph node dissection for malignant tumors.
(9) Either of the following:
  (i) The primary tumor and regional lymph node metastases are considered technically or functionally unresectable based on evaluation by a head and neck surgeon.
  (ii) Patients do not wish to undergo surgical resection despite it being deemed feasible.
(10) No dementia, or dementia with adequate family support to permit continuation of protocol treatment and evaluations.
(11) All of the following laboratory criteria:
  (i) Neutrophil count ≥1500/mm^3^
  (ii) Hemoglobin ≥9.0 g/dl
  (iii) Platelet count ≥100 000/mm^3^
  (iv) Total bilirubin ≤2.0 mg/dl
  (v) AST ≤100 U/l
  (vi) ALT ≤100 U/l
(12) Renal function: eGFR ≥50 ml/min/1.73 m^2^.
(13) Written informed consent.

### Exclusion criteria

Patients meeting any of the following criteria are excluded:

(1) Synchronous or metachronous (within 3 years) double cancers, except intramucosal tumors curable with local therapy.
(2) Active infection requiring systemic therapy.
(3) Fever >38°C.
(4) Psychological disorder that interferes with study participation.
(5) Ongoing systemic corticosteroid or immunosuppressive therapy.
(6) Uncontrolled hypertension.
(7) History of unstable angina within 3 weeks or myocardial infarction within 12 months before registration.
(8) New York Heart Association functional class ≥II or ejection fraction <50%.
(9) Diabetes mellitus with HbA1c ≥8.0%.
(10) Clinically diagnosed liver cirrhosis classified as Child–Pugh class B or C.
(11) History of cerebrovascular disease within the past 12 months, or residual deficits impairing activities of daily living or social functioning if earlier.
(12) Chronic pulmonary disease (interstitial pneumonia, pulmonary fibrosis, or severe emphysema, alone, or in combination) with chronic obstructive pulmonary disease stage III or higher.
(13) Hearing impairment of grade ≥ 3, according to CTCAE v5.0 (adults not enrolled in a monitoring program).
(14) Positive hepatitis B surface antigen or hepatitis C virus antibody.
(15) Positive HIV antibody.
(16) Inability to abstain from smoking and alcohol during protocol treatment.

### Randomization

After confirmation of eligibility, patients will be randomized in a 1:1 ratio to either the RT-alone arm or the weekly CDDP+RT arm using the web-based system at the JCOG Data Center. The minimization method with a random component will be used for randomization with the primary site and clinical stage (p16-positive oropharyngeal cancer stage II or III vs. p16-negative oropharyngeal, hypopharyngeal, or laryngeal cancer stage III vs. p16-negative oropharyngeal, hypopharyngeal, or laryngeal cancer stage IVA–IVB), ECOG PS (0 vs. 1–2), and institution.

### Treatment methods

The protocol treatment consists of RT alone or weekly CDDP+RT. Patients assigned to the weekly CDDP + RT arm will receive concurrent CRT with CDDP at 40 mg/m^2^ administered once weekly on days 1, 8, 15, 22, 29, 36, and 43 for 7 weeks. In both arms, RT will be delivered with 4–10 MV photons using intensity-modulated radiation therapy and the simultaneous integrated boost technique. A total dose of 70 Gy will be delivered to the gross tumor volume (GTV) in 2 Gy fractions once daily over 7 weeks. For the prophylactic field, a total dose of 56 Gy will be delivered in 35 fractions over 7 weeks [20, 32].

The GTV is defined as the volume of the primary tumor and the metastatic lymph nodes measuring ≥10 mm in short-axis diameter in the cervical region, ≥5 mm in the retropharyngeal region, or if smaller nodes exhibited irregular enhancement or central necrosis. A 5-mm margin is generally added to the GTV to create clinical target volume (CTV) 70 [33]. CTV56 includes the tumor-adjacent region and elective neck nodal areas. The tumor-adjacent region is defined as a 5-mm expansion around CTV70 [33]. The elective neck nodal areas include bilateral cervical lymph nodes, and details are defined according to the primary tumor site and extent of nodal involvement [34, 35].

The planning target volume (PTV) is defined as the CTV plus a 3–5 mm margin to account for organ motion and daily setup error. The dose prescription to each target will be based on D95. Organs at risk (OARs) include the brain, brainstem, spinal cord, cochlea, parotid glands, mandible, pharyngeal constrictor muscles, oral cavity, submandibular glands, larynx, and thyroid gland. Dose constraints for the PTV and OARs are specified in the protocol, and quality assurance of radiation planning is mandatory for all registered patients.

### Follow-up

All randomized patients will be followed up for at least 5 years after completion of accrual, and the primary analysis of the primary endpoint will be conducted 5 years after accrual completion. During protocol treatment, evaluations of general condition, safety, and blood tests will be performed at least every 2 weeks in the RT-alone arm and at least weekly in the weekly CDDP + RT arm.

The initial efficacy assessment will be performed 16 weeks after treatment initiation and will include contrast-enhanced CT or MRI of the head and neck, contrast-enhanced CT of the chest and abdomen, and head and neck endoscopy. If post-treatment contrast enhancement attributable to tumor shrinkage (scarring) complicates response assessment, positron emission tomography (PET)-CT will be performed 4 weeks after the initial evaluation because of the difficulty in accurately determining treatment response.

Biopsy is recommended when macroscopic residual disease is suspected.

If biopsy findings are positive, if PET-CT demonstrates fluorodeoxyglucose (FDG) uptake with corresponding lesions confirmed on CT/MRI, or if disease progression is confirmed, salvage surgery will be considered. Salvage procedures will include primary lesion excision and/or neck dissection, as appropriate.

Following initiation of protocol treatment, assessments of general condition, AEs, blood tests including thyroid function, and imaging assessments will be performed every 4 months during the first 2 years, every 6 months during years 2–3, and annually thereafter. Evaluations of QOL, IADL, and methods of nutrition support will be conducted at 16 weeks and annually for up to 5 years after treatment initiation. Geriatric assessment will be performed using IADL, the G8 screening tool, and the Charlson Comorbidity Index (CCI). The G8 and CCI will be assessed at baseline, while IADL will be evaluated both at baseline and longitudinally after treatment initiation. Methods of nutrition support (nutrition access) refer to the primary mode of nutritional management during treatment, including oral intake, enteral nutrition (e.g. gastrostomy or nasogastric tube), and parenteral nutrition. Hearing function will be assessed using pure-tone audiometry at 1, 3, and 5 years after treatment initiation. The detailed assessment schedule from the pretreatment period onward is presented in Table 1.

**Table 1:** Study calendar.

	Pretreatment	During treatment		After initiation of treatment									
		RT alone	Weekly CDDP+RT	–90D^a^	91D–2Y^b^	2Y–3Y^c^	3Y–^d^	16 W	1Y	2Y	3Y	4Y	5Y
ECOG PS	○	△^2W^	△^1W^	◎	△^4M^	△^6M^	△^1Y^						
Body weight	○	△^2W^	△^1W^										
Blood examination	○	△^2W^	△^1W^	◎	△^4M^	△^6M^	△^1Y^						
Safety profile		△^2W^	△^1W^	◎	△^4M^	△^6M^	△^1Y^						
CT/MRI	○				△^4M^	△^6M^	△^1Y^	○					
Head and neck endoscopy	○							○					
QOL	○							○	○	○	○	○	○
Geriatric assessment	○							○	○	○	○	○	○
Nutrition access	○							○	○	○	○	○	○
Pure-tone audiometry	○								○		○		○

◎: at least every 4 weeks from the end of treatment to 90 days after the start of radiotherapy.

△^2W^: every 2 weeks, △^1W^: every week, △^4M^: every 4 months, △^6M^: every 6 months, △^1Y^: every year.

ECOG PS, Eastern Cooperative Oncology Group performance status; CT, computed tomography; MRI, magnetic resonance imaging; QOL, quality of life.

^a^From end of treatment to 90 days after initiation of treatment.

^b^From 91 days to 2 years after initiation of treatment.

^c^From 2 to 3 years after initiation of treatment.

^d^From 3 years after initiation of treatment.

### Study design and statistical analysis

This randomized trial is designed to confirm the superiority of CRT with weekly CDDP over RT alone in terms of OS in elderly patients with locally advanced HNSCC who are not amenable to surgery and are unfit for CRT with high-dose CDDP. If CRT with weekly CDDP demonstrates a statistically significant improvement in OS compared with RT alone, it will be considered the new standard treatment. Conversely, if superiority is not demonstrated, RT alone will remain the standard treatment. Based on previous reports of RT alone in cohorts consisting of elderly patients or including elderly individuals, the 5-year OS in the RT-alone arm was estimated at 46% [7, 28, 36–39]. An additional improvement of 13% in 5-year OS in the weekly CDDP + RT arm is assumed [28, 29, 40, 41], corresponding to an HR of 0.679. Using the Schoenfeld and Richter method [42], the required sample size was calculated to be 240 patients (total required number of events, 145) to achieve 75% power with a one-sided alpha level of 5%, an accrual period of 5 years, and a follow-up period of 5 years. The planned total sample size was set at 250 patients to account for potential loss to follow-up. All statistical analyses will be performed at the JCOG Data Center.

### Interim analysis and monitoring

Two interim analyses are planned, with adjustment for multiplicity using the Lan–DeMets method with an O’Brien–Fleming-type alpha spending function [43]. The first interim analysis will be conducted after enrollment of half of the planned sample size. The second interim analysis will be performed after completion of patient accrual, when all randomized patients have completed protocol treatment. The Data and Safety Monitoring Committee of the JCOG will independently review the interim analysis reports, separate from the group investigators and the group statisticians. In-house monitoring will be performed every 6 months by the JCOG Data Center to evaluate and enhance study progress, data integrity, and patient safety.

## jRCT registration number

This trial has been registered in the Japan Registry of Clinical Trials as jRCTs031250688 (https://jrct.mhlw.go.jp/latest-detail/jRCTs031250688).

## Participating Institutions (from north to south)

Hokkaido University Hospital, Sapporo Medical University, Iwate Medical University, Tohoku University Hospital, Miyagi Cancer Center, Akita University, Yamagata University, University of Tsukuba, Tochigi Cancer Center, Jichi Medical University, Gunma University, Saitama Medical University International Medical Center, National Cancer Center Hospital East, Chiba University Graduate School of Medicine, National Cancer Center Hospital, Nihon University Itabashi Hospital, Tokyo Medical University Hospital, Tokyo Metropolitan Cancer and Infectious Diseases Center Komagome Hospital, National Hospital Organization Tokyo Medical Center, Keio University, Showa Medical University, Institute of Science Tokyo, Jikei University Hospital, Cancer Institute Hospital of Japanese Foundation for Cancer Research, The University of Tokyo Hospital, Juntendo University Graduate School of Medicine, Yokohama City University Hospital, Tokai University School of Medicine, St. Marianna University School of Medicine, Kanagawa Cancer Center, Niigata Cancer Center Hospital, Gifu University School of Medicine, Hamamatsu University School of Medicine, Shizuoka Cancer Center, Aichi Cancer Center Hospital, Nagoya University Hospital, Fujita Health University, Aichi Medical University, Shiga General Hospital, Kyoto University Hospital, Kyoto Prefectural University of Medicine, The University of Osaka, Osaka Metropolitan University Hospital, Kindai University Hospital, Osaka International Cancer Institute, Kansai Medical University, Kobe University Graduate School of Medicine, Kobe City Medical Center General Hospital, Hyogo Cancer Center, Nara Medical University, Okayama University Hospital, Hiroshima University Hospital, Kagawa University, National Hospital Organization Shikoku Cancer Center, Kochi University, Kyushu Cancer Center, National Hospital Organization Kyushu Medical Center, Kyushu University, Okinawa Chubu Hospital.

## References

[1] Kawakita D, Oze I, Iwasaki S., et al. Trends in the incidence of head and neck cancer by subsite between 1993 and 2015 in Japan. Cancer Med 2022;11:1553–60. 10.1002/cam4.4539. PMC8921930 35029329

[2] Cancer Registry Committee, Japan Society for Head and Neck Cancer . Report of Head and Neck Cancer Registry of Japan Clinical Statistics of Registered Patients, 2021. http://www.jshnc.umin.ne.jp/report.html.

[3] United Nations . World Population Ageing 2019: Highlights. New York: United Nations, 2019; https://www.un-ilibrary.org/content/books/9789210045537.

[4] Gatta G, Capocaccia R, Botta L. Descriptive epidemiology of the head and neck cancers in old patients. Front Oncol 2023;13:1102236. 10.3389/fonc.2023.1102236. PMC10247222 37293589

[5] Mody MD, Rocco JW, Yom SS, Haddad RI, Saba NF. Head and neck cancer. Lancet 2021;398:2289–99. 10.1016/S0140-6736(21)01550-6. 34562395

[6] Adelstein DJ, Li Y, Adams GL., et al. An intergroup phase III comparison of standard radiation therapy and two schedules of concurrent chemoradiotherapy in patients with unresectable squamous cell head and neck cancer. J Clin Oncol 2003;21:92–8. 10.1200/JCO.2003.01.008. 12506176

[7] Forastiere AA, Goepfert H, Maor M., et al. Concurrent chemotherapy and radiotherapy for organ preservation in advanced laryngeal cancer. N Engl J Med 2003;349:2091–8. 10.1056/NEJMoa031317. 14645636

[8] Pignon JP, Bourhis J, Domenge C, Designé L. Chemotherapy added to locoregional treatment for head and neck squamous-cell carcinoma: three meta-analyses of updated individual data. MACH-NC collaborative group. Meta-analysis of chemotherapy on head and neck cancer. Lancet 2000;355:949–55. 10.1016/S0140-6736(00)90011-4. 10768432

[9] Pignon JP, le Maître A, Maillard E, Bourhis J, MACH-NC Collaborative Group. Meta-analysis of chemotherapy in head and neck cancer (MACH-NC): an update on 93 randomised trials and 17,346 patients. Radiother Oncol 2009;92:4–14. 10.1016/j.radonc.2009.04.014. 19446902

[10] Lacas B, Carmel A, Landais C., et al. Meta-analysis of chemotherapy in head and neck cancer (MACH-NC): an update on 107 randomized trials and 19,805 patients, on behalf of MACH-NC Group. Radiother Oncol 2021;156:281–93. 10.1016/j.radonc.2021.01.013. PMC8386522 33515668

[11] Porceddu SV, Haddad RI. Management of elderly patients with locoregionally confined head and neck cancer. Lancet Oncol 2017;18:e274–83. 10.1016/S1470-2045(17)30229-2. 28456589

[12] Kunkler IH, Audisio R, Belkacemi Y., et al. Review of current best practice and priorities for research in radiation oncology for elderly patients with cancer: the International Society of Geriatric Oncology (SIOG) task force. Ann Oncol 2014;25:2134–46. 10.1093/annonc/mdu104. 24625455

[13] Syrigos KN, Karachalios D, Karapanagiotou EM, Nutting CM, Manolopoulos L, Harrington KJ. Head and neck cancer in the elderly: an overview on the treatment modalities. Cancer Treat Rev 2009;35:237–45. 10.1016/j.ctrv.2008.11.002. 19100689

[14] Sarris EG, Harrington KJ, Saif MW, Syrigos KN. Multimodal treatment strategies for elderly patients with head and neck cancer. Cancer Treat Rev 2014;40:465–75. 10.1016/j.ctrv.2013.10.007. 24238923

[15] Maggiore R, Zumsteg ZS, BrintzenhofeSzoc K., et al. The older adult with Locoregionally advanced head and neck squamous cell carcinoma: knowledge gaps and future direction in assessment and treatment. Int J Radiat Oncol Biol Phys 2017;98:868–83. 10.1016/j.ijrobp.2017.02.022. PMC5769962 28602414

[16] Ishii R, Ohkoshi A, Kiyota N., et al. Management of elderly patients with head and neck cancer. Jpn J Clin Oncol 2022;52:313–21. 10.1093/jjco/hyac013. 35165732

[17] Duan Z, Cai G, Li J, Chen XM. Cisplatin-induced renal toxicity in elderly people. Ther Adv Med Oncol 2020;12:1758835920923430. 10.1177/1758835920923430. PMC7238313 32489432

[18] Lee JJ, Alamleh S, Zhan LJ., et al. Clinical predictors of cisplatin chemoradiation-induced ototoxicity in HPV-positive oropharyngeal squamous cell carcinoma: a case-control study. J Otolaryngol Head Neck Surg 2024;53:19160216241248671. 10.1177/19160216241248671. PMC11282567 39056507

[19] Kelly CG . Chemotherapy: United Kingdom National Multidisciplinary Guidelines. J Laryngol Otol 2016;130:S71–4. 10.1017/S0022215116000840. PMC4873925 27841142

[20] National Comprehensive Cancer Network . NCCN Clinical Practice Guidelines in Oncology: Head and Neck Cancers. Version 2.2024. Plymouth Meeting, PA: NCCN, 2024.

[21] Oosting SF, Desideri I, Staelens D., et al. Treatment patterns in older patients with locally advanced head and neck squamous cell carcinoma: results from an EORTC led survey. J Geriatr Oncol 2021;12:1261–5. 10.1016/j.jgo.2021.05.007. 33994150

[22] Yasuda K, Kiyota N, Matsuura K., et al. Indications for chemoradiotherapy in older patients with locally advanced head and neck cancer in Japan: a questionnaire survey in the JCOG head and neck cancer study group. Front Oncol 2024;14:1441056. 10.3389/fonc.2024.1441056. PMC11750991 39845314

[23] Fukushima K, Futatsugi A, Maekawa M., et al. Comparison of cisplatin-induced nephrotoxicity between single-dose and split-dose administration to rats. Biomed Pharmacother 2022;147:112619. 10.1016/j.biopha.2022.112619. 34999374

[24] Hartmann JT, Kollmannsberger C, Kanz L, Bokemeyer C. Platinum organ toxicity and possible prevention in patients with testicular cancer. Int J Cancer 1999;83:866–9. 10.1002/(SICI)1097-0215(19991210)83:6<866::AID-IJC34>3.0.CO;2-9. 10597214

[25] Rose PG, Bundy BN, Watkins EB., et al. Concurrent cisplatin-based radiotherapy and chemotherapy for locally advanced cervical cancer. N Engl J Med 1999;340:1144–53. 10.1056/NEJM199904153401502. 10202165

[26] Kiyota N, Tahara M, Mizusawa J., et al. Weekly cisplatin plus radiation for postoperative head and neck cancer (JCOG1008): a Multicenter, noninferiority, phase II/III randomized controlled trial. J Clin Oncol 2022;40:1980–90. 10.1200/JCO.21.01293. PMC9197353 35230884

[27] Sharma MK, Bhasker S, Sehrawat A., et al. An open-label, noninferiority phase III randomized trial of weekly versus three-weekly cisplatin with radical radiotherapy in locally advanced head and neck squamous cell carcinoma (ConCERT trial). J Clin Oncol 2022;40:6004. 10.1200/JCO.2022.40.16_suppl.6004.

[28] Ghosh-Laskar S, Kalyani N, Gupta T., et al. Conventional radiotherapy versus concurrent chemoradiotherapy versus accelerated radiotherapy in locoregionally advanced carcinoma of head and neck: results of a prospective randomized trial. Head Neck 2016;38:202–7. 10.1002/hed.23865. 25224814

[29] Uchinami Y, Yasuda K, Kano S., et al. Treatment outcomes of radiotherapy with concurrent weekly cisplatin in older patients with locally advanced head and neck squamous cell carcinoma. Discov Oncol 2023;14:226. 10.1007/s12672-023-00844-7. PMC10709267 38063923

[30] Rühle A, Marschner S, Haderlein M., et al. Evaluation of concomitant systemic treatment in older adults with head and neck squamous cell carcinoma undergoing definitive radiotherapy. JAMA Netw Open 2023;6:e230090. 10.1001/jamanetworkopen.2023.0090. PMC9941890 36808242

[31] Rühle A, Weymann M, Behrens M., et al. A multicenter evaluation of different chemotherapy regimens in older adults with head and neck squamous cell carcinoma undergoing definitive chemoradiation. Int J Radiat Oncol Biol Phys 2024;118:1282–93. 10.1016/j.ijrobp.2023.10.025. 37914144

[32] Yokota T, Zenda S, Kodaira T., et al. Novel approach of prophylactic radiation to reduce toxicities comparing 2-step40 with 56-Gy simultaneous integrated boost intensity-modulated radiation therapy for locally advanced squamous cell carcinoma of the head and neck, an intergroup phase III trial (JCOG1912, NEW BRIDGE). BMC Cancer 2023;23:1068. 10.1186/s12885-023-11503-z. PMC10626703 37932681

[33] Grégoire V, Evans M, Le QT., et al. Delineation of the primary tumour clinical target volumes (CTV-P) in laryngeal, hypopharyngeal, oropharyngeal and oral cavity squamous cell carcinoma: AIRO, CACA, DAHANCA, EORTC, GEORCC, GORTEC, HKNPCSG, HNCIG, IAG-KHT, LPRHHT, NCIC CTG, NCRI, NRG Oncology, PHNS, SBRT, SOMERA, SRO, SSHNO, TROG consensus guidelines. Radiother Oncol 2018;126:3–24. 10.1016/j.radonc.2017.10.016. 29180076

[34] Grégoire V, Ang K, Budach W., et al. Delineation of the neck node levels for head and neck tumors: a 2013 update. DAHANCA, EORTC, HKNPCSG, NCIC CTG, NCRI, RTOG, TROG consensus guidelines. Radiother Oncol 2014;110:172–81. 10.1016/j.radonc.2013.10.010. 24183870

[35] Biau J, Lapeyre M, Troussier I., et al. Selection of lymph node target volumes for definitive head and neck radiation therapy: a 2019 update. Radiother Oncol 2019;134:1–9. 10.1016/j.radonc.2019.01.018. 31005201

[36] Yasuda K, Uchinami Y, Kano S., et al. Radiotherapy with or without chemotherapy for locally advanced head and neck cancer in elderly patients: analysis of the Head and Neck Cancer Registry of Japan. Int J Clin Oncol 2024;29:241–7. 10.1007/s10147-023-02450-7. 38155239

[37] Forastiere AA, Zhang Q, Weber RS., et al. Long-term results of RTOG 91-11: a comparison of three nonsurgical treatment strategies to preserve the larynx in patients with locally advanced larynx cancer. J Clin Oncol 2013;31:845–52. 10.1200/JCO.2012.43.6097. PMC3577950 23182993

[38] Zandberg DP, Cullen K, Bentzen SM, Goloubeva OG. Definitive radiation with concurrent cetuximab vs. radiation with or without concurrent cytotoxic chemotherapy in older patients with squamous cell carcinoma of the head and neck: analysis of the SEER-medicare linked database. Oral Oncol 2018;86:132–40. 10.1016/j.oraloncology.2018.09.023. 30409293

[39] Saunders MI, Rojas AM, Parmar MK, Dische S, CHART Trial Collaborators. Mature results of a randomized trial of accelerated hyperfractionated versus conventional radiotherapy in head-and-neck cancer. Int J Radiat Oncol Biol Phys 2010;77:3–8. 10.1016/j.ijrobp.2009.04.082. 20394851

[40] Sakashita T, Homma A, Hatakeyama H., et al. Clinical outcomes of weekly cisplatin chemoradiotherapy for patients with pyriform sinus cancer. Int J Clin Oncol 2015;20:1081–5. 10.1007/s10147-015-0821-8. 25804347

[41] Buglione M, Alterio D, Maddalo M., et al. Three weekly versus weekly concurrent cisplatin: safety propensity score analysis on 166 head and neck cancer patients. Radiat Oncol 2021;16:239. 10.1186/s13014-021-01966-4. PMC8686550 34930353

[42] Schoenfeld DA, Richter JR. Nomograms for calculating the number of patients needed for a clinical trial with survival as an endpoint. Biometrics 1982;38:163–70. 10.2307/2530299. 7082758

[43] Lan KKG, DeMets DL. Discrete sequential boundaries for clinical trials. Biometrika 1983;70:659–63. 10.2307/2336502.

